# Analectic

**Published:** 1829-03

**Authors:** 


### MISCELLANEOUS INTELLIGENCE.

### I.—Analectic.

1. *Blisters in the early stage of Measles.*—The *Lancet* of 27th September, contains the following communication from Mr. Matthews, member of the Royal College of Surgeons.

"I have had extensive opportunities of witnessing a great many cases of Measles with its attendants, viz. inflammation of the lungs, and continued chronic cough, and have seen it treated according to common principles, with variable success. I am therefore induced to recommend a plan of treatment which, for its simplicity and utility, I think, cannot be surpassed, having more than a hundred times adopted it, and with a success which has entirely exceeded my most sanguine expectations. I am, therefore, convinced, that the disease may be cut considerably shorter, and many valuable lives saved, to the comfort of numerous families. I do not mean to put it forth as a novelty, but so far as I have any knowledge, it has not been employed in the manner I am about to mention. It is simply this—as soon as the first symptoms of the complaint show themselves, such as sneezing with defluxions from the nose and eyes, *and before the eruption appears*, the prompt application of a blister to the chest, (of course with other suitable remedies,) seems to act like a charm, in most cases entirely preventing any affection of the lungs supervening. I have lately treated three cases, two according to the plan already laid down, and one upon the common principles of our best physcians, the parents of the child being averse to blisters. The latter is now scarcely recovered, after being ill six weeks; the two former were perfectly recovered in a fortnight. I shall not attempt to explain the modus operandi of blistering, early, but the conclusions, I think, to be drawn, are these:—

1. That the disease is rendered shorter and milder; 2. That the disposition to inflammation of the lungs, and its consequences, if not entirely prevented, is very much mitigated; 3. That the patients are not more predisposed to colds or coughs, than others in whom no disease had taken place; and, 4th. That the recovery is more rapid and lasting."—*Month. Jour. of For. Med. No.* 13.

2. *Mr. Lizars' Method of Amputation.*–In amputation of the thigh, arm, and fore-arm, Mr. Lizars makes two flaps, cutting from without inwards, with the knife the same length as the longest used by Lisfranc, with this difference, however, that it has only one cutting edge. In amputation of the thigh, he makes the outer flap first, cutting nearly traversely to the bone, the incision pointing a little obliquely upwards towards the trunk; he then carries it along the outer edge of the bone, four or five inches, in the same direction according to the muscularity of the limb. The inner flap is made to correspond precisely with the outer; the assistant instantly grasping the inner flap, and thus completely commanding the artery; so that, if the operator chooses, he may with safety perform the operation without a tourniquet.

The superiority of this mode of amputation over that of transfixion, is said to be, that the surgeon can lengthen his flaps as much as he pleases, even in the middle of the operation, if he considers them too short to cover the bore; besides, he cuts in the same line of direction; whereas, in transfixion, he cuts first from, and then towards himself. In this mode also the flaps are much fuller and thicker; and no lower third flap can possibly remain as in transfixion.—*Ibid.*

3. *Mercury.*—M. Colson, surgeon to the Hotel Dieu de Noyon, who has long been engaged in investigating the effects of mercury upon the system, has recently succeeded in detecting the presence of this mineral in the blood of persons to whom it had been administered. In one instance, a young man had swallowed, by mistake, four or five ounces of the liquor of Van Sweiten; violent fever supervened, requiring venesection. Furnished with a plate of burnished brass, M. Colson directed the jet of blood upon it, and left it in the basin for the space of twenty-four hours, at the end of which time, mercurial stains upon the brass became evident. A similar result was obtained in the other instances. Fourcroy relates in his translation of Ramazzini, that the serum having been collected from a great number of phlyctenæ, which appeared upon the thighs and legs of a gilder, an infinite number of mercurial globules was found in the bottom of the vessel containing it. M. Gaspard has found them in the alvine evacuations; Petronius in the matter ejected by vomiting, where the mineral had been employed in frictions; Rhodius, Breyer, Valvasor, Guidot, Vercelloni, Burghard, Didier, Haeschtter, and recently Dr. Cantu of Turin, in the urine. It has been found in the bones, synovial membranes, in the pleura, and in the lungs. In our own day, M. Dumeril has found it in different parts of the body; MM. Orfila and Pickel have obtained it by distillation of the substance of the brain and nerves: and it is stated in the last number of the *Bull. des Sciences Medicales*, that Professor Hunefield of Griefswold, has found it in its metallic state in the semi-liquid contents of a lipoma.—*Ibid.*

4. *Treatment of Dropsies.*—The September number of the *Revue Medicale* contains some observations by M. Guibert on the application of the *methode iatraleptique* to the treatment of dropsies, and particularly of ascites.

This method consists in the employment, two or three times a day, of frictions with the following liniment upon the thorax, abdomen, or legs, according to the seat of the disease. Tincture of squills of digitalis, and of colchicum seeds, each half an ounce; camphorated and ammoniated oil, an ounce and a half. The frictions to be made with a woollen or flannel cloth, and to be continued from five to twenty minutes, according to the quantity of fluid extravasated, and the urgency of the symptoms. Diuretic beverages, and pills composed of squills, digitalis, &c. are at the same time given internally. This treatment will, of course, be varied according to circumstances; it may be sometimes advisable to suspend it for a time, or to conjoin it with demulcents, gentle laxatives, enemata, &c. &c. and if the dropsy be recent, and the cause producing it be known, its removal by appropriate means should constitute the first indication.

The *methode iatraleptique*, observes M. Guibert, such as I employ it in the treatment of dropsies, has especially appeared to me very advantageous in ascites; and it is principally to the use of the liniment above mentioned, that I attribute the success which I have met with in the cure of that disease. The remedies given internally, have been highly useful as auxiliaries, but alone, were insufficient to produce the profuse urinary discharges, I might almost say, the artificial diabetes, which I have observed in different cases, and the rapid disappearance of the great extravasations which I have had to treat. A number of cases, corroborative of the efficacy of the plan, is related, from which M. Guibert deduces the following conclusions:—

1. The cases above mentioned, and I might adduce many others, evidently prove that diuretics, when opportunely administered, and in sufficiently large doses, are, of all the remedies used in dropsy, those which are most uniformly successful.

2. The iatraleptic method, or the use of frictions, is one of the most advantageous modes of employing these medicines, inasmuch as they thus act upon a large surface, and do not fatigue the stomach.

3. It is, nevertheless, always useful to associate with it internal remedies, in order to obtain a more prompt effect.

4. This combined treatment is sufficiently active to produce, in a very short space of time, the diminution of a dropsy which has reached a considerable volume, and thus to obviate, in many cases, the necessity of paracentesis; an operation almost always merely palliative, and which must be repeatedly performed, at intervals more or less protracted, in order to prolong the existence of the patient; whereas, by the persevering employment of the plan which I have advised, we may expect to produce the complete absorption of the effused fluid.

5. Finally, this method is attended with no inconvenience, provided the inflammatory symptoms, if any exist, have been previously removed by appropriate means. In cases complicated with organic alterations, it will even succeed in exciting the absorbent and urinary systems, although the primitive cause of the disease still exists, and sooner or later must occasion its re-appearance. In simple ascites on the contrary, and in anasarca, or œdema of the lower extremities, its success is almost infallible.—*Ibid.*

5. *Puerperal Insanity.*—From 57 cases of this malady treated by Dr. Burrows, he deduces the following corollaries:

1. That mania is a more frequent consequence of lying-in, and the process of lactation, than any other variety of mental derangement.

2. That puerperal insanity occurs from the age of twenty to thirty in the proportion nearly of two to one, at all other ages.

3. That in London, physical causes much more frequently originate puerperal insanity than moral causes, the physical being to the moral as ten to one.

4. That the access of puerperal insanity happens before the fourteenth day in three out of five cases.

5. That it happens between the fourteenth and twenty-eighth days, in one out of about six cases and a half.

6. That nearly four in five recover their intellects.

7. That not more than half recover in six months.

8. That those recover soonest whose delirium supervenes on the process lactation.

9. That the maniacal form ceases sooner than the melancholic.

10. That the mortality is *apparently* but not *really* double Esquirol's return;* and that the greater number of deaths occurred before the second week from delivery.

11. That half, and possibly more, if the truth could always be discovered, attacked by puerperal insanity, prove to possess an hereditary predisposition.—*Commentaries on Insanity.—Ibid.*

6. *Case of Tetanus, with Inflammation of the Spinal Chord, and disease of the Anterior Roots of the Spinal Nerves.*—This case, which occurred at Udina, is strongly in favor of the opinion, that the cause of tetanus is inflammation of the spinal chord; it also confirms Mr. Bell's idea, that movement depends on the anterior, and sensation on the posterior, roots of the spinal nerves.

A woman, of 40 years of age, felt, in consequence of over exer-

*There were only six deaths in 92 cases recorded by Esquirol, and as not one of them occurred till more than six months from the access of the insanity, Dr. Burrows attributes the difference to their having all become chronic, and taken in the aggregate he observes, they do not give a greater rate of mortality, than is allotted to the same number who are insane from other causes. Of the 57 cases mentioned above, ten terminated fatally.

tion a difficulty of moving the lower jaw, a stiffness of the neck, and a tensive pain in the limbs. On the ninth day, after the first appearance of these symptoms, she was taken to the hospital; tetanus and trismus were then fully developed: the former in the form of emprosthotonos. Warm baths seemed to diminish the spasmodic affection of the jaw, but that of the trunk increased, and carried the patient off on the twelfth day. On examination after death, the brain was found in a healthy state; the vertebral canal was filled with a bloody serum; the anterior portion of the spinal chord was of a yellowish dirty white colour, and covered with small, round, and oval hydatids, from the size of a millet seed to that of a pea; its substance exhibited reddish spots; the posterior part was healthy; the posterior roots of the spinal nerves had a very different appearance from the anterior roots; the latter were evidently softened, and presented a yellow colour; the former were perfectly healthy.—*Lancet.*

7. *Difference of the Blood in the Veins and Capillary Vessels.*— The October number of the *Journal de Chimie Medicale*, contains some experiments by Dr. Pallas, which go to establish what might *a priori*, have been supposed, but what, previously to the researches of this gentleman, had not been the subject of direct experiment, namely, that the blood in the capillary system, is more highly animalized than that contained in the veins, being heavier, of a brighter colour, more odorous and viscous. The following is an abstract of the experiment referred to.

A man who had had tertian fever for six days, during the greater part of which time he had been strictly dieted, was bled from the arm, while, at the same time, twelve leeches were applied on the right, and three cups on the left side of the epigastrium. A vessel filled with the venous blood as it flowed from the arm, weighed 19,-950 grammes; the same vessel filled with the blood drawn by leeches, weighed 20,450 grammes; an equal quantity taken by means of cupping weighed 20,400. The venous blood had a deep black colour, and separated in a few hours into crassamentum and serum; that drawn by the leeches, was more viscous, presented a bright red color, and exhaled an odour resembling that of a mixture of bile and urine; the crassamentum was larger than that formed from the venous blood. The blood obtained by means of the sacrificator, had a deep red colour, was viscous, and exhaled a well marked bilious odour. The serum of the three kinds of blood was clear and transparent; that which was furnished by the blood drawn by the leeches, was red, and of a deeper color than the others. Treated separately with an ounce of distilled water, the three specimens were subjected to ebulition to coagulate the albumen, in order to appreciate more correctly the proportion of the liquid to the solid parts. The following was the result obtained. Solid parts of the venous blood well dried, 2,550; of the blood drawn by leeches, 3,100; of the blood obtained by cupping, 3,000.

The subject of the second experiment had had, for several days an intermittent gastric affection, accompanied with general irritation of the vascular system. The vessel above mentioned filled with blood as it flowed from the vein, weighed 20,350; an equal quantity taken, not as in the preceding experiment, from that which the leeches had sucked, but collected after their fall from the bites which they had made, weighed 20,750: in this instance also, the latter was heavier, more coloured, odorous, and viscous than the former. Treated with water as before, the venous blood gave of solid parts 2,550; that taken from the capillary vessels, 2,630.

In a third experiment, which was accidentally interrupted, the blood drawn from the vein, and from the capillary system, weighed respectively 20,700, and 20,950; as in the other experiments, the latter was also more highly coloured, more odorous, and viscous than the former.

It is to this difference of chemical composition, observes M. Pallas, that we must attribute the preference given by physicians to local bleeding in a variety of cases. Although many causes must concur to vary the proportions of the constituent principles of human blood, such as age, sex, temperament, constitution, manner of living, health, disease, &c., &c.; the difference which we have pointed out must be uniform in the same person, and explains the important rank accorded to the blood of the capillary vessels, in the production of the phenomena of life.—*Month. Jour. For. Med. No.* 14.

8. *Cure of White Swelling by Frictions of Iodine.* By Dr. Lugol.—The use of iodine in scrofulous tumours is strongly recommended by the most eminent French surgeons. M. Breschet, in his lectures, speaks of it in the highest terms. The same treatment is pursued with advantage at the Hospital St. Louis, from the records of which a recent cure of white swelling and tumour of the jaw may be cited as a proof of its efficacy.

The patient had white swelling, with fistulous ulcers on the knee, the leg was bent on the thigh, and utterly useless. He had also a large tubercular tumour on the right side of the face, which seems to have its origin over the maxillary joint. The swelling was such that the man could scarcely open his mouth, and the flat edge of a penny-piece was the largest substance he could introduce between his teeth. These tumours have entirely disappeared under the use of Iodine frictions.—*Jour. de Hopitaux.—Ibid. No.* 15.

9. *Extirpation of the Uterus.*—This operation has been performed under different circumstances, which require to be distinguished, to prevent the results in one class of cases from influencing our estimate of the operation in cases which are dissimilar.

1st. Oslander performed the excision of the neck of the uterus, and M. Lisfranc has repeated it so often, that there can be no doubt of its being in many instances an operation without pain or danger.

No one, however, who knows the subject accurately, can read these cases without seeing that the operation was in the great number of instances unnecessary, from the absence of malignant disease, while in those cases in which such disease was present it was unsuccessful. What is called cancer of the uterus is seldom or never so confined to its neck that the removal of this part will remove the disease; and all that these operations prove is, that the operators know little of the disease which they imagine they are extirpating, and that the excision of the neck of the uterus is not attended with so much danger as might have been anticipated. Let not, however, the impunity with which the neck of the uterus has been removed, be confounded with the result of extirpating the whole organ—they are entirely different operations; and what is true of the former, throws no light on the latter.

2dly. Cancer has occurred conjoined with a complete collapse of the uterus, so that the whole organ has protruded externally, and has been removed by a ligature round the inverted vagina, above the tumour. As this compound case occurs very rarely, this operation very seldom admits of being performed.

3dly. The inverted uterus has been removed successfully by ligature. In this operation it is the fundus and part of the body only, which are removed; the rest of the body and neck, through the orifice of which they had protruded, being left behind.

4thly. The whole uterus, in its natural situation, has been removed in a considerable number of cases on the continent, and lately by Dr. Blundell in this country. A month or two ago this enterprising practitioner favoured us with the details of a successful case, which has produced a strong sensation among the practitioners of England. We learn, on unquestionable authority, that he has since then performed the operation again, and that the patient died, (it is said of hemorrhage,) within six hours afterwards. We know, on equally good authority that he had performed the operation some time before the successful case, and that the patient died soon after in a state of collapse, without any hemorrhage to explain it. Of the three operations, therefore, which Dr. Blundell has performed, only one has been successful,—the two others being followed by the speedy death of the patients.

In our last Gazette we published a case in which this operation was performed by Mr. Banner, the surgeon of Liverpool; and in which the woman died on the fourth day after the operation. We applaud the conduct of Mr. Banner on this occasion—not merely for the dexterity with which he seems to have operated, but for the courage and candour with which he comes forward and publishes an unsuccessful case.

The extirpation of the whole uterus has now been performed in England four times within the two last years; three times by Dr. Blundell, and once by Mr. Banner of Liverpool: of these cases one only has recovered; the three others have lost their lives. It is important this should be known, that those who venture on the operation may be fully acquainted with the slight chance which it affords of success. The extirpation of the whole uterus may be called the forlorn hope of surgery—a kind of lover's leap; in which most of those who take it perish in the attempt, but those few who escape alive are cured.

We sincerely hope, and fully expect, that Dr. Blundell, having published his solitary case of success, will publish also his two unsuccessful cases: for on such a subject, not only the truth, but the whole truth ought to be told, in order to guide the profession right in so difficult and important a question. It may be some time before he has leisure to draw up an account of the particulars; in the meanwhile this general statement may supply its place, and is especially necessary, because cases are continually occurring in which the propriety of performing the operation will have to be discussed, and which renders an accurate estimate of its dangers of the utmost value. Let it be remembered that out of four cases in which the uterus has been extirpated in England three have proved fatal.*—*Lon. Med. Gaz.*

10. *On the Coagulation of the Blood*—Dr. John Davy relates a number of interesting experiments on this subject in the *Edinburgh Medical and Surgical Journal*, for October, 1828. The following are his conclusions. 1st. The coagulation of the blood takes place independent of motion, and it is very little affected by motion, whether violent or moderate; 2dly. That it is not caused by the action of atmospheric air, or much affected by any kind of air that is not absorbable in water; 3dly. That it is not retarded by the introduction and absorption of carbonic acid gas; and lastly, That the action of reagents on the blood, or the fibrin of the blood, as regards coagulation, is exceedingly various not to be anticipated *a priori*, and inexplicable on any one hypothesis hitherto advanced, the effect of each of them requiring special consideration, and further and minute experimental enquiry for its elucidation.—*Am. Jour. Med. & Phy. Sciences. No.* 6.

11. *On the Diseases of the Kidneys and Ureters.* By J. Bouillaud, M. D.—Dr. B. has frequently met with *hypertrophy of the kidneys*, it generally occurs only in one of these organs. It is recognized, according to Dr. B. by the following appearances. The kidney is a quarter, or a third, or perhaps even one half, larger than the natural size. Its substance is firmer, more compact, and redder. It is probable that in such cases the renal artery is enlarged, although this fact has not been determined. Hypertrophy of the kidney occurs under the influence of various causes, which determine to it an unusual quantity of blood. The most likely circumstance to produce this kind of plethora in one kidney is the exist-

*Since writing the above, we have heard that the operation has been performed in Edinburgh, by Mr. Lizars.

ence of some obstruction to the passage of the blood towards the other. It happens, consequently, that hypertrophy of one kidney is frequently detected when the other is in a state of atrophy. Hypertrophy of the heart and of the external muscles, take place equally under the same conditions which preside over the increased size of the kidney.

*Atrophy, or diminished nutrition of the kidneys.*—This disease M. B. has frequently seen. Its characters are diametrically opposite to those of hypertrophy of the organ. The size of the kidneys is less than natural; their substance is paler; they contain less blood and appear shrunk. Whatever cause obstructs the current of blood to the kidneys, may produce an atrophy of them. In every such instance, M. B. has been able to demonstrate a greater or less obstruction to the free circulation of the blood. The pressure of an enlarged spleen has sometimes produced atrophy of the left kidney.—The right kidney has been similarly affected by the continued, yet gradual, pressure of an ealarged liver. In other organs as the heart, the lungs, the breast, the testcle, pressure frequently causes the same diminution of size.

*Infiltration of urine, and cysts of the kidneys.*—M. B. observes that no pathologist has hitherto described this affection; it is not, however, very rare, but it may easily escape the observation of a careless practitioner; the following are the characters of it: on the surface of the kidneys may be seen several round vesicles, which raise the covering membrane of these organs. These vesicles appear to be small cysts in the substance of the kidney, and are probably formed by a certain quantity of urine, which has distended the uriniferous tubes, in consequence of some obstruction to the passage of the fluid. M. B. has seen some of these cysts as large as a cherry. Sometimes, instead of numerous vesicles, he has detached one large sac, which he presumed to have been formed from the union of several smaller ones, of which the parietes had ruptured. He has found the whole of the kidney transformed into one large sac, containing either a transparent serous or turbid fluid.

*Inflammation of the kidneys, and of the disorganization which follow inflammation.*—In consequence of their peculiar structure, the kidneys do not easily become the seat of those disorganizations which result from inflammation. Nephritis is marked by the following appearances: redness, tumefaction, presence of pus, softening of the structure of the organ, abscesses, ulceration of the external surface, conversion of the parenchymatous substance into a tuberculous or encephaloid matter, which is in a great measure, the product of the diseased secretion of the affected kidney. Cysts, either on the surface, or in the substance of the kidney, may result from inflammation. In two or three cases, M. B. has found the kidney converted into a fatty yellowish substance. The symptoms of the various ulcerations which the kidneys occasionally undergo, are very obscure; this circumstance will not be considered so extraordinary when we consider, first, that the deep-seated situation of the kidney embarasses our examinations; secondly, that derangement of the function of the kidneys produces similar symptoms to those which result from various affections of the bladder and ureters; thirdly, that pain is by no means a constant attendant upon renal disease. If we are to rely upon the statement of most pathologists, acute pain is the almost inseparable attendant upon inflammation of the kidneys; it is not denied that such is frequently the case, but M. B. affirms that he has observed the most decided marks of renal inflammation in the bodies of patients, who had never complained of pain in the region of the kidneys. This absence of pain may be more easily conceived, when we reflect that the kidneys in a natural state are but slightly sensible. Violent pain is not seldom complained of in the region of the kidneys, when no disease of them is to be detected. The presence of a certain quantity of blood or pus in the urine, when there exists no disease of the bladder, is a symptom of some affection of the kidney; when to this symptom is united a smart attack of fever, the existence of nephitis may be strongly presumed. At the commencement of the disease, if both kidneys are affected, an almost total suppression of urine takes place. Chronic nephritis, like most other internal inflammations of a chronic character, produces a slow fever, which destroys the patient by throwing him into that state termed renal consumption. When the effected kidney continues the performance of its functions, the urine is much altered in its appearance, but sometimes it ceases to secrete; the urine being formed only by the healthy kidney, presents no unusual appearance, and the diagnosis of the disease is then extremely difficult. If both kidneys are simultaneously disorganised, so that a total cessation of the secretion of urine takes place, the same phenomena will occur as we observe in animals in which both ureters are tied, or both kidneys removed; violent fevers quickly arise, and a strong smell of urine is exhaled from the body. Is hypertrophy of the kidneys ever the cause of diabetes? M. B. is not furnished with sufficient facts to justify him in giving a positive answer to this question, but he has observed hypertrophy of the kidneys where the patient had been affected with diabetes. The ureters, like all other parts of the body, may suffer from inflammation, and undergo various alterations of structure in consequence; their canals may be much enlarged, diminished or entirely obliterated; dilatation of the ureter may arise from any cause which obstructs the free passage of the urine into the bladder; contraction or obliteration may follow from any accidental compression, from inflammation of the internal membrane which lines the cavity, or from the cessation of the passage of the urine through the canal, from the function of the kidney being no longer performed in consequence of disease. The symptoms of affections of the ureters are as obscure as those which attend diseases of the kidneys. If both canals are obliterated at the same time, death would speedily result; but if one uterer only is obstructed, the calibre of the liver will be increased considerably, from the additional duty which it will have to perform under such circumstances. In support of these observation, M. Bouillaud details several interesting cases.—*Journal Complementaire, July*, 1828.—*Ibid.*

12. *On the Use of Circular Ligatures in Intermittents.*--About thirty years ago the application of the tourniquet was recommended by Mr. Kellie, a naval surgeon, as a preventive of the paroxysm of intermittent fever, and several instances are recorded by him in Duncan's Medical Commentaries, for 1794, in which he used it with success. He applied the instrument to the arm of one side, and the thigh of the other. Some further experiments have recently been made by Dr. Robouam, on the anti-febrile powers of circular ligatures, and the result is favorable. The ligatures were applied to the arm, and made sufficiently tight to interrupt the superficial circulation, and to retard that of the more deep-seated vessels. As soon as the extremities began to redden, the patient felt easier, and the symptoms of the approaching paroxysm abated; the cold and trembling ceased; the pulse became more free, &c. Nearly in all the cases Dr. R. found two or three applications of the ligature were sufficient to suppress the fever.

According to Dr. R. the following rules are to be observed: 1st. The most favorable time for the application of circular ligature is the beginning of the cold stage, when they seldom fail to suppress or mitigate the fit. They have much less effect in the middle of the paroxysm, and none at all during the intermission. 2d. In case of syncope, the ligatures must be loosened; the patient usually bears them better after some time. 3d. If the ligatures are too tight, they may produce dangerous symptoms, and even suffocation. 4th. The application of circular ligatures has an effect corresponding to blood-letting.—*Ibid.*

13. *On certain methods of treating Chronic Inflammations of the Eye, lately adopted at the Royal Westminster Ophthalmic Hospital.* By G. J. Guthrie.—Mr. Guthrie says that he has been employing with advantage the following remedies in chronic ophthalmia:—

1. ℞. Argenti Nitratis, gr. ij. ad gr. x.; Liq. Plumbi Subacet. gtt. xv.; Ung. Cetacei ℥j.

2. ℞. Hydr. Oxymur. gr. iij. ad iv.; Liq. Plumbi Subacet. gtt. xv.; Ung. Cetacei ℥j.

The argentum nitratum and oxymuriate of mercury must be reduced first to an impalpable powder, then mixed with the ointment on a slab, and the liquor plumbi added. It may be done in a glass mortar. A double decomposition takes place in either ointment, which naturally diminishes the strength of each; but this change takes place slowly, particularly in the oxymuriate ointment, so that weeks elapse before they become inert. A very sensible difference is felt by the patient between an ointment only two days made, and another of two or three weeks standing, and the stimulating qualities may be calculated according to the state of the eye, as well as the strength of the composition. The argentum nitratum ointment is gray when first made, but soon changes its colour to a brownish-black. If the argentum nitratum be mixed with the ung. cetacei, (as I once used it,) without the liquor plumbi, it dissolves more rapidly; when used, the powdered nitrate falls into the fold of the conjunctiva, or rests on the lid, and is apt to cause a slough, which is prevented by adding the lead.

The manner of using the ointment is "by introducing between the lids a portion, larger or smaller, as the case may seem to require it, from the size of a large pin's head to that of a garden pea. The eye lids being closed, are to be rubbed gently with the finger, so as to diffuse the dissolving ointment over the whole surface of the conjunctiva; a part of it usually, however, works out by the motion of the lids, and should be wiped off, (if the nitrate of silver,) to prevent its staining the skin. Both ointments cause pain: in some persons it is considerable, in others less so, lasting from half an hour to an hour and a half; and when the ointment is newly made, sometimes for four hours, and even until the next day. On the subsidence of the pain caused by the ointment, that which previously existed is found to be relieved, if not entirely removed; and on the subsequent day, the patient usually acknowledges the benefit he has received, with regard to all the symptoms. When the application has been severe, and the patient very irritable, a state resembling white chemosis, occasionally takes place, and appears formidable to a person unacquainted with the effect of the remedy; it soon, however, subsides. The eye should be fomented with warm anodyne fomentations.'

Mr. G. rarely repeats the application until the third day, 'but the feelings of the patient are the best guide, the return of some of the old sensations indicating the necessity for its use, which should be, if possible, anticipated. In some cases of acute inflammation, two or three applications will arrest the progress of a serious disease, and effect a cure. In chronic cases, the ointment must be continued for a considerable time, and occasionally alternated with other remedies. Where they create a state of regularly increasing irritation, as they sometimes will do, cupping, purgatives, &c. are of service; when the remedies may again be resorted to.'

Several cases are related by Mr. G. in which these applications appear to have been strikingly beneficial.—*Lond. Med. and Phys. Journ. Sept.* 1828.—*Ibid.*

14. *Remedy for opacity of the cornea.*—The following combination is recommended in the *Journal de Chimie Medicale, &c. for September*, 1828, as an useful application to promote the absorption of the effused lymph in opacity of the cornea. ℞. oxyd. hydrar., rub., agaric., alb. āā ʒss. Sacch. alb., ʒj. misce intim. et pulv. subtil. A small portion to be blown into the eye daily.—*Ibid.*

15. *Signs of Drowning.*—M. Orfila has made a great number of experiments with the view of determining whether submersion has taken place prior or subsequent to death—an important decision in legal medicine. The following are the inferences which he has drawn.

1. That the red, livid, and swelled condition of the face, with froth at the mouth and nostrils, which some authors have laid down as indicating that the submersion has taken place during life, leads to no such inference, as it is wanting in many who have been drowned, and is present in many who have met their death by other means.

2. That the same remarks apply to extreme paleness of the face which is an effect of the body remaining long in the water. M. Orfila here describes the alterations which the skin undergoes in those who have been long submersed. He asserts than on the legs the integuments become indigo colour, and then brownish on exposure to the air, while the rest of the body is very white, but the moment it comes in contact with the air, it is successively converted into brown and green, commencing at the chest. Remaining long in the water also brings on abrasions of the skin, which may give rise to the idea of wounds having been inflicted.

3. Excoriations of the fingers and traces of dirt under the nails are of no assistance, because they are wanting in those who are drowned before they come to the bottom, while they may be present in a body which, being thrown into a river strikes against va ious obstacles.

4. Injections of the brain and its membranes, M. Orfila thinks would be a satisfactory indication of drowning, if it were proved that the body became cold in a vertical position. As, however, this sign is frequently absent in those who have been drowned, so it cannot be regarded as a positive proof.

5. In those who have been drowned, the right cavities of the heart, the venæ cavæ, the pulmonary veins and arteries, are generally distended by a quantity of black blood, while the left side of the heart and the aorta are much less filled: the right ventricle is of a blackish brown; the left of a clear rose colour; and the right cavities retain a less contractile power than the left. This condition, however, is met with in many cases of sudden death.

6. Although the blood is generally fluid, yet M. Orfila has seen it coagulated in one individual who was drowned: a fact also observed by Lafosse, and more recently by M. Avissard.

7. The dissection of more than fifty persons who had been drowned, has satisfied M. Orfila that it is a mistake to suppose that in drowning the individuals die during inspiration, and that, in consequence, they have the diaphragm pushed into the abomen, and the chest elevated.

8. He regards the colour of the abdominal viscera as indicative of asphyxia in general, but not of drowning in particular.

9. The experiments of Orfila, and some other physiologists, show that water enters the stomachs of those who drown themselves; while this is not the case with regard to bodies thrown into the water.

But, in order to give its full value to this sign, it would require to be proved that the water had neither been swallowed before death, nor injected after it.

10. It is not true that the epiglottis is pushed down upon the larynx.

11. Great importance had been attributed to the presence of sanguineous froth in the windpipe. M. Orfila objects, however, that this exists in other cases, as in death from epilepsy and hanging; while it is wanting in the drowned who have remained long under water, without coming to the surface to breathe.

M. Orfila made a considerable number of experiments to ascertain if water enters into the ramifications of the bronchia of the drowned; and he has always found that the fluid entered the lungs of the animal, and that the quantity was greater or less in proportion to the care took to keep the head erect in lifting the animal out of the water. But the fluid is not found in the air-passages, if the examination be made soon after death. As to the question, whether the water enters the lungs after death, the experiments of M. Orfila agree with those of M. Piorry, proving that it does enter, and more completely the more vertical the position of the animal; and consequently, the presence of water is not a sufficient proof of drowning, as water enters the air-passages of the dead body, and that the only absolute sign is the presence of water in the minute ramifications of the bronchia: it being proved that this water is the same as that in which the individual was immersed, that it had been injected after death, and could not have penetrated to the lungs from the vertical position of the body in the liquid."—*Med. Chir. Rev. Aug.* 1828.

